# Comparison of three different sedative-anaesthetic protocols (ketamine, ketamine-medetomidine and alphaxalone) in common marmosets (*Callithrix jacchus*)

**DOI:** 10.1186/1746-6148-9-113

**Published:** 2013-06-11

**Authors:** Jaco Bakker, Joost J Uilenreef, Eva RJ Pelt, Herbert PM Brok, Edmond J Remarque, Jan AM Langermans

**Affiliations:** 1 Animal Science Department, Biomedical Primate Research Centre, Lange Kleiweg 161, 2288 GJ, Rijswijk, The Netherlands; 2 Department of Clinical Sciences of Companion Animals, Faculty of Veterinary Medicine, University of Utrecht, Yalelaan 106, 3584 CM, Utrecht, The Netherlands; 3 Department of Parasitology, Biomedical Primate Research Centre, Lange Kleiweg 161, 2288 GJ, Rijswijk, The Netherlands

**Keywords:** Alphaxalone, Atipamezole, Common marmoset, Immobilisation, Induction, Ketamine, Medetomidine, Recovery, Sedation

## Abstract

**Background:**

Handling of common marmoset (*Callithrix jacchus*) usually requires chemical restraint. Ketamine has been associated with muscle damage in primates, while common marmosets, compared to other primates, additionally display an exceptional high sensitivity to ketamine-associated side-effects. Notably, muscle twitching movements of limbs and hands, and a marked increase in salivation are observed. We investigated two alternative intramuscular (i.m.) immobilisation protocols against ketamine (50 mg/kg; protocol 1) in a double-blind randomised crossover study in ten healthy adult common marmosets for use as a safe reliable, short-term immobilisation and sedation. These protocols comprised: alphaxalone (12 mg/kg; protocol 2) and 25 mg/kg ketamine combined with 0.50 mg/kg medetomidine (reversal with 2.5 mg/kg atipamezole; protocol 3A). Following completion and unblinding, the project was extended with an additional protocol (3B), comprising 25 mg/kg ketamine combined with 0.05 mg/kg medetomidine (reversal with 0.25 mg/kg atipamezole, twice with 35 min interval).

**Results:**

All protocols in this study provided rapid onset (induction times <5 min) of immobilisation and sedation. Duration of immobilisation was 31.23 ± 22.39 min, 53.72 ± 13.08 min, 19.73 ± 5.74 min, and 22.78 ± 22.37 min for protocol 1, 2, 3A, and 3B, respectively. Recovery times were 135.84 ± 39.19 min, 55.79 ± 11.02 min, 405.46 ± 29.81 min, and 291.91 ± 80.34 min, respectively. Regarding the quality, and reliability (judged by pedal withdrawal reflex, palpebral reflex and muscle tension) of all protocols, protocol 2 was the most optimal. Monitored vital parameters were within clinically acceptable limits during all protocols and there were no fatalities. Indication of muscle damage as assessed by AST, LDH and CK values was most prominent elevated in protocol 1, 3A, and 3B.

**Conclusions:**

We conclude that intramuscular administration of 12 mg/kg alphaxalone to common marmosets is preferred over other protocols studied. Protocol 2 resulted in at least comparable immobilisation quality with acceptable and less frequent side effects and superior recovery quality. In all protocols, supportive therapy, such as external heat support, remains mandatory. Notably, an unacceptable long recovery period in both ketamine/medetomidine protocols (subsequently reversed with atipamezole) was observed, showing that α-2 adrenoreceptor agonists in the used dose and dosing regime is not the first choice for sedation in common marmosets in a standard research setting.

## Background

The common marmoset (*Callithrix jacchus*) is frequently used in biomedical research [1,2]. Although certain procedures can sometimes be performed without sedation, more complex procedures require immobilisation. However, limited information is available in the literature on marmoset chemical restraints [3]. The use of inhalation techniques is impractical when dealing with large numbers of animals simultaneously in environments without an operating theatre. Therefore, ketamine is commonly used for immobilisation, either alone or combined with other sedatives and/or analgesics [3-5]. The main disadvantages of ketamine are poor muscle relaxation, grasping movements of limbs and hands, and a marked increase in salivation [4,6]. In addition, ketamine has been associated with muscle damage in primates [7-9].

Alternatively, sedation with alphaxalone-alphadolone (Saffan®, Althesin®) was recommended for marmosets [3,5,10]. Despite the relatively large injection volume, no muscle damage was observed [5]. Alphaxalone-alphadolone was solubilised with Cremophor®-EL, which resulted in adverse effects in dogs, cats, and humans [11,12] and consequently it was withdrawn from the market. Recently, a new formulation of alphaxalone without alphadalone and solubilised with the aid of cyclodextrin rather than Cremophor®-EL became available (Alfaxan®). This is a short-acting, injectable anaesthetic agent for the induction and maintenance of general anaesthesia in dogs and cats [13-15]. Its clinical off-label administration has been described in several species, including ponies, pigs, and goats [16-22].

A short-term anaesthesia regimen frequently used in dogs and cats is ketamine combined with medetomidine [23,24]. Concurrent use of medetomidine reduces the amount of ketamine required, induces additional analgesia as well as curtailing increased muscle tone and salivation. An important advantage is the quick recovery to normal function following reversal of medetomidine with the specific antagonist atipamezole in dogs and cats [23-26]. In addition, the combination ketamine/medetomidine caused markedly less damage to muscle tissue at injection sites than did the single use of ketamine in rats [27]. The use of ketamine/medetomidine has been described in several primate species [27-35], but these reports offer little guidance related to its practical implementation in the common marmoset.

Due to the lack of knowledge on safe, reliable and short-term sedation protocols in the common marmoset, we designed a crossover study to assess and compare the effects of ketamine, ketamine-medetomidine (reversed with atipamezole) and alphaxalone administered i.m. for allowing the undertaking of minor invasive procedures such as blood collection from the femoral vein, tuberculin testing, routine veterinary interventions such as minor surgical procedures or wound care, radiography, and ultrasonography.

## Methods

### Animals, housing, and care

Ten (5 male and 5 female) healthy adult common marmosets *(Callithrix jacchus)*, age 3.2 ± 1.4 years and mean bodyweight of 364 ± 27 g all originated from the Biomedical Primate Research Centre (BPRC, Rijswijk, The Netherlands). All monkeys received a complete physical, haematological, and biochemical examination before the study started. Only animals with all values within the normal range were included. They remained under intensive veterinary supervision during the entire study period. Animals were housed with a same-sex buddy in spacious cages (150 × 75 × 185 cm) enriched with branches and toys, in compliance with the new EU directive 63/2010. The animals were fed commercial monkey pellets (Ssniff®, Soest, Germany) supplemented with Arabic gum and limited amounts of fresh fruit. Drinking water was available *ad libitum.* Room temperature was 23.2-26.8°C, with a 12 hour light:dark cycle. Food was removed 16 hours prior to sedation but water intake was never restricted.

### Ethical consideration

The experimental protocol (DEC-BPRC number: # 665) was approved by the Animal Experiments Committee (DEC) of the BPRC. The DEC based its decision on ‘De Wet op de Dierproeven’ (The Dutch ‘Experiments on Animals Act’, 1996) and on the ‘Dierproevenbesluit’ (The Dutch ‘Experiments on Animals Decision’, 1996). Both documents are available online at http://wetten.overheid.nl.

### Experimental design and drug administration

The study was initially performed in three sessions, with a 28-day washout interval in a prospective, double blinded crossover design. Each animal was randomised to receive one of the following sedation protocols: (1) Ketamine hydrochloride (Ketamine 10%; Alfasan Nederland BV, Woerden, NL, 100 mg/ml), at a dose of 50 mg/kg ketamine i.m.; (2) alphaxalone (Alfaxan; Vetoquinol B.V., ‘s-Hertogenbosch, NL, 10 mg/ml), at a dose of 12 mg/kg alphaxalone i.m.; (3A) 25 mg/kg ketamine, and medetomidine hydrochloride (Sedastart; AST Farma B.V., Oudewater, NL, 1 mg/ml), at a dose of 0.5 mg/kg i.m. combined in the same syringe just before its use, reversed with atipamezole hydrochloride (Sedastop; AST Farma B.V., Oudewater, NL, 5 mg/ml), at a dose of 2.5 mg/kg atipamezole i.m. after 10 min of immobilisation. To maintain a double-blind design and to exclude any effect of mechanical damage from the amount of fluid i.m. injected, saline (sodium chloride 0,9%; B. Braun Medical B.V., Oss, NL) was added to ascertain that all volumes injected were identical. Animals that did not receive atipamezole received an equal volume of saline, 10 min after immobilisation was recorded.

Twenty eight days after the third sedative session (day 112), following unblinding of the dataset, it was decided to add an additional sedation round (protocol 3B) with ketamine (25 mg/kg) combined with a tenfold lower medetomidine dose (0.05 mg/kg) followed by atipamezole injection of 0.25 mg/kg after 10 min and similar amount 35 min later if the recovery phase had not ended.

While it is standard to use sedation when handling common marmosets, for the purpose of this study we avoided this procedure for bodyweight measurement and blood sampling because unsedated control values to determine the effect of the sedation were required.

Prior to each sedation, and one and two days following sedation, bodyweight was registered to determine the correct dosage of the sedative, and to determine the possible effect of sedation on bodyweight development. Marmosets were trained to voluntarily enter a Perspex cylinder and were taken out of their cage by means of this cylinder. The cylinder with the animal in it was placed on a weighing scale as a non-invasive means of assessing the bodyweight.

For injection, one person manually restrained the animal while a second person administered the anaesthetic volume i.m. into the quadriceps muscle mass on the anterior thigh, into the left or right quadriceps femoris, using a 26G needle. Care was taken that the drugs were not injected directly into the circulation.

After injection of the sedative, each marmoset was released into its home cage, where it was monitored until immobilisation was achieved. Once immobilised, the animals were taken to an adjacent quiet, temperature-controlled room (24°C) for measurements. Procedures were carried out using routine practice standards for short minimally invasive procedures, without supportive care (i.e. external heat or secured airway). The marmosets were breathing room-air spontaneously throughout the experiment. When the immobilisation period ended, the animals recovered in their home cage on an open non-heated blanket, facilitating visual control by the observer during the recovery period.

### Determination of induction, immobilisation and recovery characteristics

For each sedation event, induction, immobilisation, and recovery times were recorded. Induction time was defined as the time between injection and loss of postural tonicity. Immobilisation time was defined as the time from the loss of postural tonicity to the animal’s first attempt to lift its head. Recovery time was defined as the time from the animal’s first attempt to lift its head until the moment that the marmoset could walk and climb confidently in the restricted confines of its cage and could be reunited safely with its companion. Total procedure time was defined as the sum of induction time, immobilisation time, and recovery time.

For each sedation event, the quality of induction and recovery as a whole and at 3-min intervals during immobilisation were scored on an ordinal scale by a treatment-blinded observer (Table 1). The reliability of immobilisation was judged by the degree of muscular tension (Table 2) and pedal withdrawal reflex (Table 3), while sedation was judged by the palpebral reflex (Table 4) [36]. To allow for a meaningful comparison between groups, immobilisation quality- and reflex-scores were censured from statistical analysis after 9 minutes. Only descriptive statistics were performed on the immobilisation quality data and reflex scores from 9 minutes into the immobilisation onwards.

**Table 1:** Scorecard for quality of induction, immobilisation and recovery

**Score**	**Quality**	**Character**
1	Good	No vocalisation, salivation, compulsive licking or sneezing. No increased attention towards injection site, no involuntary/uncoordinated muscle activity
2	Satisfactory	Some vocalisation and/or involuntary/uncoordinated muscle activity, salivation, compulsive licking, sneezing, some discomfort from injection (< 5 min)
3	Unsatisfactory	Violent struggling/no immobilisation effectuated, severe discomfort from injection (increased attention towards injection site > 5 min), excessive salivation, vomiting, compulsive licking, sneezing, involuntary muscle activity

**Table 2:** Scorecard for muscular tension

**Score**	**Quality**	**Character**
0	No muscle tension	Complete relaxation, adequate muscle relaxation for performing minor invasive procedures
1	Normal muscle tension	Partial relaxation
2	Increased muscle tension	Rigidity in muscles

The muscular tension was assessed by resistance to flexion of limbs (leg muscle tension was evaluated by flexion and extension of the left rear leg) and by opening the jaws (jaw tension was evaluated by pulling the lower jaw open by an index finger).

**Table 3:** Scorecard for the pedal withdrawal reflex

**Score**	**Quality**	**Character**
0	No reflex	There was no increased muscle tension and/or bending of the knee for at least one second after removing the haemostat
1	Normal reflex	There was muscle tension and/or bending of the knee
2	Increased reflex	There was increased muscle tension, bending of the knee, and muscle vibrations/involuntary movements of other limbs

The pedal withdrawal reflex was determined by applying a haemostat (Hartman baby mosquito BH104R, straight, 100 mm, 4″) on the first clip for 1 second on phalanx III, just above the nail of the left rear leg.

**Table 4:** Scorecard for the palpebral reflex

**Score**	**Quality**	**Character**
0	No reflex	No narrowing of the eyelids or muscle movement
1	Moderate reflex	Delayed and/or incomplete closing of the eyelids
2	Normal reflex	The eyelids immediately close fully

The palpebral reflex was tested by lightly touching the medial canthus of the left eye with a dry cotton swab without touching the cornea.

During immobilisation, pulse rate (PR), indirect systolic, diastolic, and mean arterial blood pressure (SAP, DAP, and MAP, respectively) were recorded at 3-min intervals using a non-invasive oscillometric device (vetHDO monitor with MDSoftware, using a Criticon®Soft-cuf®, size: I, colour: white) at the base of the tail. Respiratory rate (RR) was determined by observing thoracic excursions over a 30 second period and multiplied by two. Pulse haemoglobin oxygen saturation (SpO_2_) was measured using an earlobe clip of a veterinary pulse oximeter (Ohmeda biox 3740; BOC Health Care, Inc., Louisville, USA) applied to the right hand. Rectal body temperature was monitored at 3-min intervals using a digital thermometer (Microlife®Vet-temp; Microlife®, Widnau, Switzerland) with a measurement range of 32°C to 42.9°C. To allow for a meaningful comparison between groups, PR, MAP, RR, SpO_2,_ and rectal body temperature were censured from statistical analysis after 9 minutes.

### Blood sampling

To determine possible local myotoxic effects of the injected formulations, levels of AST, LDH, and CK were determined in serum. Samples (200 μl blood) were taken from unsedated animals prior to administration of the sedative and 24 and 48 h post dosing. Control samples from unsedated animals were collected on day 0, 1, 2, 140, 141, and 142. Samples were collected by one person restraining the animal while a second person performed the blood sampling by inserting a needle (26 gauge) percutaneously into the *vena saphena*, confirmed by self-filling of the needle tip with blood, of which blood was collected for testing. Afterwards, firm pressure was applied to the sample site for 2 min to minimise haemorrhage rises. The samples were processed immediately with a Cobas Integra® 400 plus (F. Hoffmann-La Roche Ltd, Basel, Switzerland).

### Statistical analysis

All statistical analyses were performed with the R language and environment for statistical computing (R Foundation for Statistical Computing, Vienna, Austria. ISBN 3-900051-07-0, URL http://www.R-project.org). To determine statistical significance in induction, immobilisation, and recovery times, paired t-tests were performed for the six pair-wise comparisons (P1 versus P2, P1 versus P3A, P1 versus P3B, P2 versus P3B, P2 versus P3B and P3A versus P3B). The quality of the sedation phases was analysed using the non-parametric Wilcoxon’s signed rank test. To adjust for multiple tests a Bonferroni correction was applied: the p value for statistical significance was set at 0.05/6 = 0.00833. Clinical chemistry values (AST, LDH and CK) and body weight were analysed with mixed linear models and ensuing parameters estimates are presented with 95% confidence intervals, p values of <0.05 were considered statistically significant.

## Results

### Duration and quality of the sedative protocols

**Figure 1:**
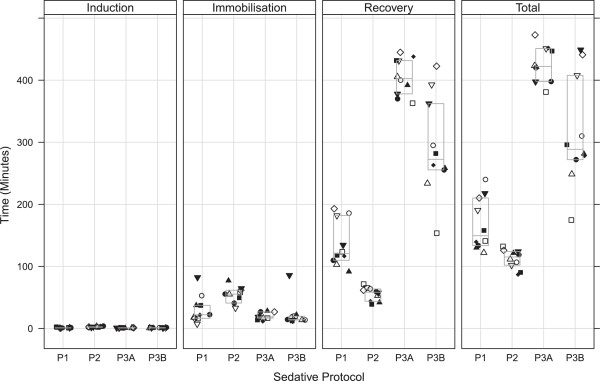
**Induction, immobilisation, recovery and total procedure time in minutes, in each sedative protocol per individual.** P1 = protocol 1, P2 = protocol 2, P3A = protocol 3A, and P3B = protocol 3B. Each symbol represents an individual animal throughout all panels. Top of the box indicates upper quartile, middle is median and bottom is lower quartile.

Induction, immobilisation, recovery and total procedure time are shown in Figure 1 and Table 5. Induction time of protocol 2 was significantly longer than that for protocol 1 (*p* = 0.0016, paired *t*-test), protocol 3A (*p* < 0.0005, paired *t*-test), and protocol 3B (*p* = 0.0001, paired *t*-test). There was no significant difference in the other comparisons. Immobilisation time in protocol 2 was of a longer duration than protocol 1, but failed to achieve statistical significance (*p* = 0.011). Immobilisation times in protocol 2 were significantly longer than those observed for protocols 3A and 3B (*p* = 0.0001 and 0.0012, respectively), both with administration of atipamezole after 10 min of immobilisation. Recovery times for protocol 1 were significantly shorter than protocol 3A and 3B (*p* values < 0.0001, paired *t*-test). Protocol 2 showed statistically significant shorter recovery times then the other protocols (*p* < 0.0001, paired *t*-test, for all comparisons). There was a significant difference in the recovery times between protocols 3A and 3B (*p* = 0.0005, paired *t*-test). In protocol 3A and 3B, a relatively long (at least 1 hour) period of apathy was observed in all monkeys after they had initially sat upright. During this time, the marmosets clung to the wire of their cage, or to branches, and remained there for prolonged periods, during which they did not react to any external stimuli.

**Table 5:** Induction, immobilisation, recovery, and total procedure time in min (mean ± SD) in each sedative protocol

	**Protocol 1**	**Protocol 2**	**Protocol 3A**	**Protocol 3B**
Induction time	1.24 ± 0.55	2.54 ± 0.65	1.17 ± 0.42	1.20 ± 0.31
Immobilisation time	31.23 ± 22.39	53.72 ± 13.08	19.73 ± 5.74	22.78 ± 22.37
Recovery time	135.84 ± 39.19	55.79 ± 11.02	405.46 ± 29.81	291.91 ± 80.34
Total procedure time	168.32 ± 45.60	112.05 ± 15.30	426.37 ± 29.11	315.89 ± 88.95

### Quality assessments

**Figure 2:**
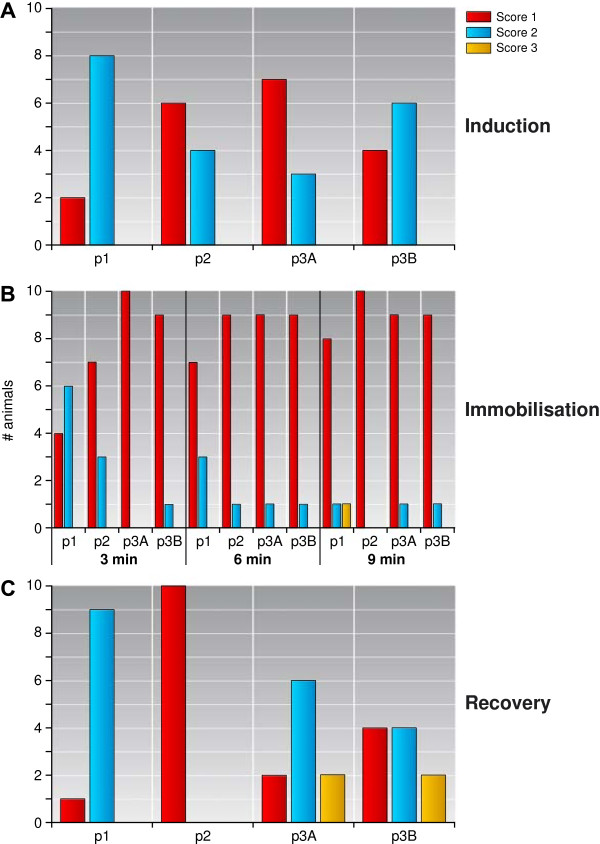
**Quality of induction, immobilisation, and recovery, scored in each sedative protocol. A** = induction, **B** = immobilisation, **C** = recovery. p1 = protocol 1, p2 = protocol 2, p3A = protocol 3A, and p3B = protocol 3B.

Quality of induction and recovery as a whole and of immobilisation at 3, 6, and 9 min are shown in Figure 2.

Quality of induction never reached the unsatisfactory score of 3 in any sedation protocols used. In protocol 1, 8 monkeys were given a score of 2 (2 due to vocalisation and salivation, 2 due to vocalization alone, and 4 due to salivation alone). In protocol 2, 4 marmosets scored a 2 (vocalisation during injection). During protocol 3A, 3 animals scored a 2 (vocalisation only); whereas in protocol 3B, a total of 6 animals scored a 2 (4 due to salivation, 1 monkeys due to salivation and vocalisation and 1 animal due to vocalisation during injection).

The quality of immobilisation was measured every 3 min for all animals during immobilisation. Because in protocol 1 some animals already started to recover after 9 min, detailed comparison of all groups was only possible for the first 9 min (Figure 2). The quality of immobilisation after 9 min is described below. In protocol 1, two animals scored a 3 while immobilised with ketamine, due to periods of apnoea, combined with involuntary limb movement. Five monkeys scored a 2 due to an observation of salivation or involuntary muscle movement. In one of those animals, quiet vocalisation and muscle twitching was observed. Three animals were given a score of 1. In protocol 2, no salivation was observed, but all marmosets displayed muscle twitches near the end of immobilisation (score 2). In protocol 3A, no marmoset was given a score of 3, while only two animals scored a 2 due to salivation or involuntary muscle movement. In protocol 3B, however, one marmoset scored a 3 again (same animal showed this also with protocol 1). A total of four marmosets reached a score of 2 due to salivation or involuntary muscle movement. Eight and five animals for group 3A and 3B respectively were given a score of 1.

Quality of recovery was given a score of 1 for one animal for group 1, all animals for group 2 and two and four animals for group 3A and 3B respectively. Nine animals sedated with ketamine scored a 2 due to salivation. With protocol 3A, six marmosets scored a 2, and with protocol 3B, four animals were given a score of 2, all because of excessive salivation. In both protocols 3A and 3B, two marmosets scored a 3 due to excessive salivating and vomiting. The quality of recovery was significantly lower in protocol 1 as compared to protocol 2 (*p* = 0.006, Wilcoxon signed rank test).

### Physiological parameters

During immobilisation, cardiorespiratory parameters were continuously scored with 3-min interval. The 3, 6, and 9 min data are shown in Table 6. No significant differences were observed for these parameters between the used protocols. RR, SpO_2_, PR, and MAP values scored during the total immobilisation period were generally within clinically acceptable limits during all protocols and there were no fatalities. However, in protocol 1, two animals experienced a short period of apnoea. In protocol 3B, one marmoset (same animal showed this also with protocol 1) experienced again an apnoea period. No cyanosis of the visible mucous membranes was observed.

**Table 6:** Cardiorespiratory parameters averages and standard deviation values

	**3 min**	**6 min**	**9 min**
P1 PR	367 ± 36*	371 ± 23	348 ± 50*
P2 PR	330 ± 52	320 ± 51	310 ± 51
P3A PR	236 ± 35	230 ± 32	221 ± 19
P3B PR	263 ± 29*	257 ± 18	237 ± 15
P1 MAP	87 ± 8	91 ± 17	85 ± 16*
P2 MAP	94 ± 21	94 ± 24	96 ± 28
P3A MAP	75 ± 11	64 ± ±6	63 ± 8
P3B MAP	96 ± 24	87 ± 21	89 ± 25
P1 Body Temp	38.8 ± 0.5	38.4 ± 0.5	37.8 ± 0.4*
P2 Body Temp	38.9 ± 0.5	38,6 ± 0.4	38.3 ± 0.5
P3A Body Temp	38.8 ± 0.2	38,2 ± 0.3	37.8 ± 0.3
P3B Body Temp	39.2 ± 0.4	38.9 ± 0.4	38.5 ± 0.4
P1 SpO_2,_	86 ± 6	86 ± 5	86 ± 15*
P2 SpO_2,_	80 ± 9	83 ± 10	80 ± 11
P3A SpO_2,_	93 ± 3	92 ± 3	93 ± 3
P3B SpO_2,_	86 ± 7	84 ± 7	84 ± 8
P1 RR	58 ± 17	56 ± 10	58 ± 14*
P2 RR	41 ± 10	38 ± 9	38 ± 6
P3A RR	61 ± 18	56 ± 14	57 ± 10
P3B RR	58 ± 12	59 ± 13	59 ± 14

PR (beats/min), MAP (mm Hg), body temperature (°C), SpO_2_ (per cent)_,_ and RR (per min) in the tested sedation protocols during the first 9 min of the immobilisation period, with 3 min interval. *P1* protocol 1, *P2* protocol 2, *P3A* protocol 3A, and *P3B* protocol 3B. n = 10 unless specified, *n = 9.

In all sedation protocols, monkeys were between 38–39.5°C at the beginning of each procedure; the rectal body temperature dropped by a total of 3°C within 20 min. Temperature measurements taken during recovery tended to show a progressive decrease, even below 32°C, until normal activity was resumed.

No effects of the atipamezole injection(s) on physiological parameters were observed.

### Reliability

**Figure 3:**
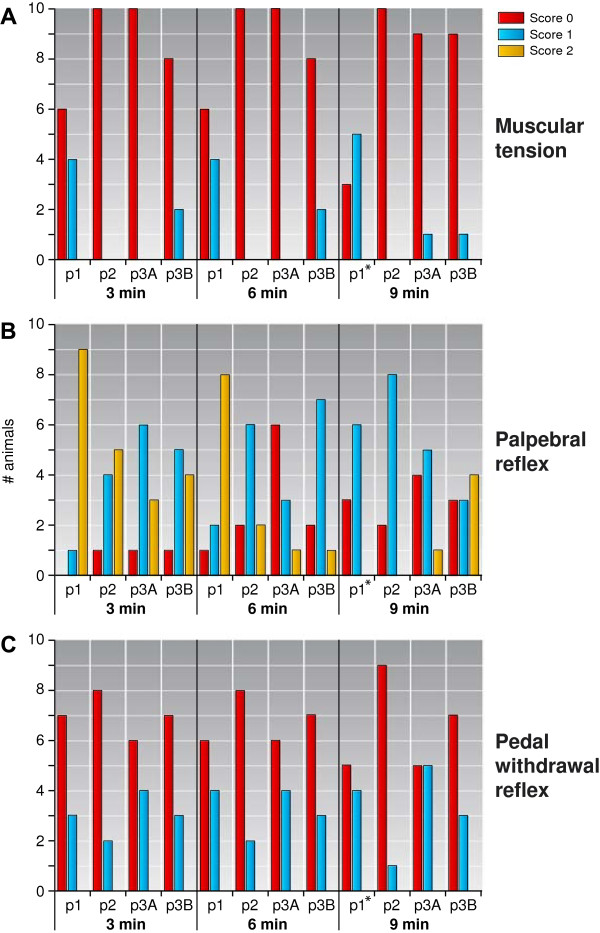
**Reliability in the tested sedation protocols during the first 9 min of the immobilisation period, with 3 min interval (A) Muscular tension (B) palpebral reflex (C) pedal withdrawal reflex.** p1 = protocol 1, p2 = protocol 2, p3A = protocol 3A, and p3B = protocol 3B. n = 10 unless specified; *n = 9.

For all monkeys muscle tension, palpebral reflex and withdrawal reflex were continuously scored with 3-min interval. The 3, 6, and 9 min data are shown in Figure 3A, B, and C.

The data of the whole immobilisation period was afterwards judged and is represented in Table 7. For the muscle tension, data protocol 1: five out of the eight that did not reach a score of 0 never reached a score of 1 either. Data protocol 2: all animals’ muscle tension scores were initially 0 and increased to a score of 1 towards the end of the immobilisation period. Data protocol 3A/B: Other protocols differed in scores between 0 and 1, with only a few marmosets reaching a score of 2.

**Table 7:** The reliability in total of the tested sedation protocols during the immobilisation

	**Palpebral reflex**				**Withdrawal reflex**				**Muscle tension**			
	P1	P2	P3A	P3B	P1	P2	P3A	P3B	P1	P2	P3A	P3B
Good	2	7	6	3	7	10	8	8	6	10	9	9
Not Good	8	3	4	7	3	0	2	2	4	0	1	1

*P1* protocol 1, *P2* protocol 2, *P3A* protocol 3A, and *P3B* protocol 3B. The reliability was judged by the degree of muscular tension, pedal withdrawal reflex, and palpebral reflex and was afterwards divided into ‘good’ and ‘not good’. The number of animals, which reached at least the palpebral score of 0 were defined as ‘good’ and the animals that never reached a score lower than 1 as ‘not good’. The number of animals reaching a withdrawal score of 0 was judged as ‘good’ and animals never reaching a score lower than 1 were judged as ‘not good’. The number of animals that reached a muscle tension score of maximum 1 was judged as ‘good’ and animals that reached a score of 2 were judged as ‘not good’.

The palpebral reflex scores differed markedly in time and between individuals, with some animals never achieving a score of 0. All animals under all sedative protocols used achieved a score of 2 before the end of immobilisation. Three animals in protocol 2 did not reach a score of 0, but were all given a score of 1 at several points during the observations. Only one of the four animals in protocol 3A that did not reach a score of 0 was never given a score of 1. Two of the seven animals in protocol 3B did not reach a score of 0 were never given a score of 1.

Regarding the withdrawal reflex, no score of 2 was given to any animal during any of the sedations. Several animals never reached a score of 0, with the exception of protocol 2, as all these animals reached a score of 0.

### Assessment of muscle damage

**Figure 4:**
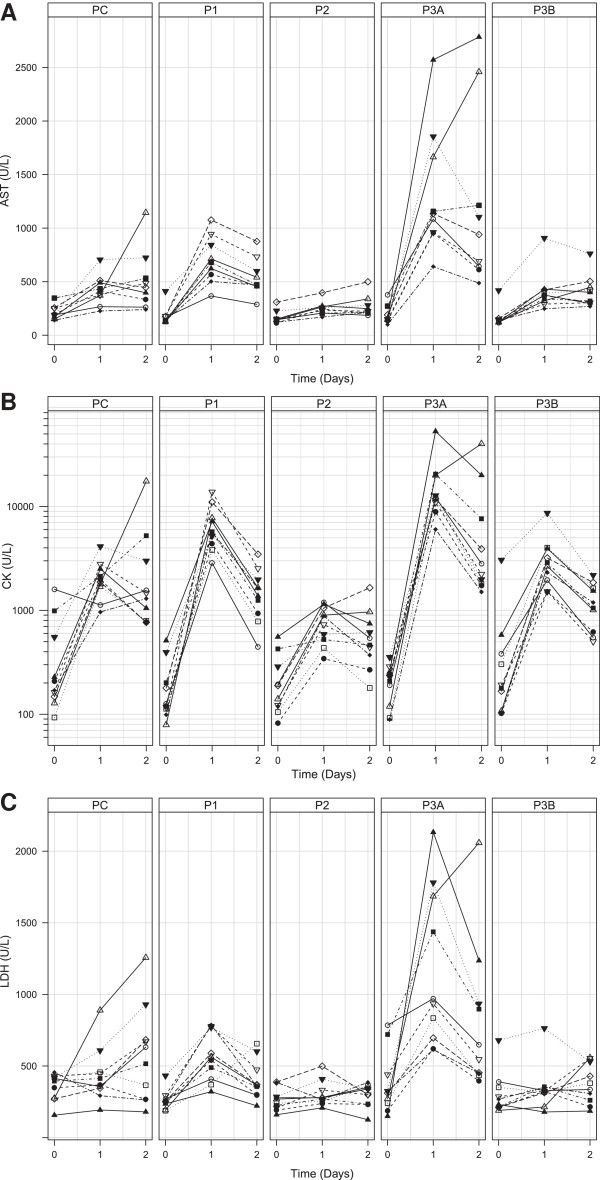
**Blood values of each sedative protocol (A) AST, (B) CK and (C) LDH.** Data presented per individual at day 0, 1, and 2. Separate panes represent the sedation protocols (PC = control protocol, P1 = Protocol 1, P2 = Protocol 2, P3A = Protocol 3A, P3B = Protocol 3B). Each symbol represents an individual animal throughout all panels.

The first day after sedation, AST levels (Figure 4A) had increased significantly for protocols 1, 3A and 3B as well as for the control protocol (all p < 0.05; Table 8). A small but statistically non-significant increase in AST levels was observed for protocol 2 (Table 8). AST levels remained elevated on the second day after sedation for protocols 1 and 3 as well as for the control protocol (all p < 0.05; Table 8). A statistically non-significant increase in AST levels was observed for protocols 2 and 3B (Table 8).

**Table 8:** Estimated changes in muscle damage indicators (AST, LDH, and CK) and body weight one and two days after procedures

**Day 1**						
**AST**	**Est (95% CI)**	**PC**	**P1**	**P2**	**P3A**	**P3B**
PC	198.8 (8.5 to 389.1)		**	NS	***	NS
P1	526.0 (335.7 to 716.3)	**		***	***	*
P2	79.4 (−110.9 to 269.7)	NS	***		***	NS
P3A	1123.2 (932.9 to 1313.5)	***	***	***		***
P3B	246.4 (56.1 to 436.7)	NS	*	NS	***	
LDH	Est (95% CI)	PC	P1	P2	P3A	P3B
PC	78.5 (−119.6 to 276.6)		NS	NS	***	NS
P1	299.2 (101.1 to 497.3)	NS		NS	***	NS
P2	40.0 (−158.1 to 238.1)	NS	NS		***	NS
P3A	795.4 (597.3 to 993.5)	***	***	***		***
P3B	43.3 (−154.8 to 241.4)	NS	NS	NS	***	
CK	Est (95% CI)	PC	P1	P2	P3A	P3B
PC	7.2 (4.0 to 12.9)		***	NS	***	NS
P1	37.8 (21.1 to 67.7)	***		***	NS	**
P2	3.9 (2.2 to 7.0)	NS	***		***	*
P3A	74.2 (41.5 to 132.8)	***	NS	***		***
P3B	11.0 (6.2 to 19.7)	NS	**	*	***	
Body Weight	Est (95% CI)	PC	P1	P2	P3A	P3B
PC	−0.9 (−4.5 to 2.7)		NS	NS	**	NS
P1	0.5 (−3.1 to 4.1)	NS		NS	***	*
P2	1.8 (−1.8 to 5.4)	NS	NS		***	**
P3A	−8.5 (−12.1 to −4.9)	**	***	***		NS
P3B	−5.1 (−8.7 to −1.5)	NS	*	**	NS	
Day 2						
AST	Est (95% CI)	PC	P1	P2	P3A	P3B
PC	286.0 (34.2 to 537.8)		NS	NS	***	NS
P1	362.9 (111.1 to 614.7)	NS		NS	***	NS
P2	96.8 (−155.0 to 348.6)	NS	NS		***	NS
P3A	1019.7 (767.9 to 1271.5)	***	***	***		***
P3B	239.8 (−11.9 to 491.6)	NS	NS	NS	***	
LDH	Est (95% CI)	PC	P1	P2	P3A	P3B
PC	217.8 (13.7 to 421.9)		NS	NS	NS	NS
P1	137.8 (−66.3 to 341.9)	NS		NS	*	NS
P2	33.9 (−170.2 to 238.0)	NS	NS		**	NS
P3A	430.1 (226.0 to 634.2)	NS	*	**		**
P3B	72.2 (−131.9 to 276.3)	NS	NS	NS	**	
CK	Est (95% CI)	PC	P1	P2	P3A	P3B
PC	6.8 (3.3 to 13.8)		NS	NS	*	NS
P1	8.6 (4.2 to 17.6)	NS		*	NS	NS
P2	2.8 (1.4 to 5.8)	NS	*		***	NS
P3A	21.9 (10.7 to 44.8)	*	NS	***		***
P3B	4.2 (2.0 to 8.5)	NS	NS	NS	***	
Body Weight	Est (95% CI)	PC	P1	P2	P3A	P3B
PC	0.2 (−3.9 to 4.3)		NS	NS	NS	NS
P1	1.9 (−2.2 to 6.0)	NS		NS	NS	*
P2	2.1 (−2.0 to 6.2)	NS	NS		*	*
P3A	−2.4 (−6.5 to 1.7)	NS	NS	*		NS
P3B	−3.5 (−7.6 to 0.6)	NS	*	*	NS	

*PC* control values, *P1* protocol 1, *P2* protocol 2, *P3A* protocol 3A, and *P3B* protocol 3B. Changes in AST, LDH and body weight are presented as day X – day 0 values; changes in CK are presented as fold increases day X/day 0 values (95% CI). The columns labelled PC, P1, P2, P3A and P3B summarise between treatment differences (NS = not significant, * < 0.05, ** < 0.01, *** < 0.001).

The first day after sedation, LDH levels (Figure 4B) had increased significantly for protocols 1 and 3A (all p < 0.05; Table 8). A small but statistically non-significant increase in LDH levels was observed for protocols 2 and 3B (Table 8). The increase in LDH levels continued for animals in the control group and protocol 3A; day 2 LDH levels were significantly elevated as compared to day 0 (Table 8). LDH levels remained elevated in protocols 1, 2 and 3B but this failed to reach statistical significance (Table 8).

CK levels increased significantly one day after sedation in all protocols as well as in the control protocol (Table 8) (Figure 4C). The increase in CK levels was significantly higher in protocols 1 and 3 as compared to controls or protocol 2 (Table 8). CK levels decreased slightly as compared to day 1, but remained elevated on the second day after sedation in all groups (Table 8).

### Body weight

**Figure 5:**
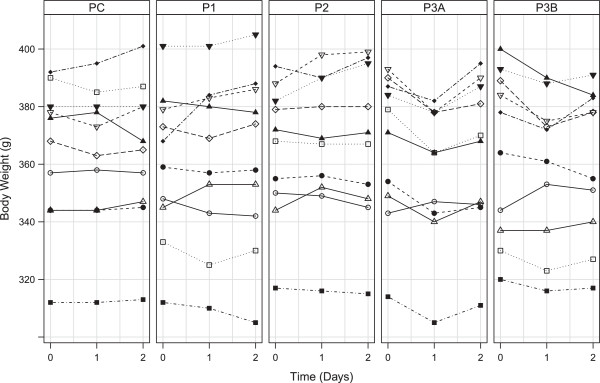
**Body Weight values of each sedative protocol.** Data presented per individual at day 0, 1, and 2. Separate panes represent the sedation protocols (C = control protocol, P1 = Protocol 1, P2 = Protocol 2, P3A = Protocol 3A, P3B = Protocol 3B).

The first day after sedation body weight had significantly dropped for protocol 3A, whilst non-significant changes were observed for the control protocol and protocols 1, 2 and 3B (Table 8; Figure 5). The second day after sedation body weights did not differ significantly from baseline values for any of the protocols under investigation.

## Discussion

This is the first study that directly compared the effect of various sedation protocols, including alphaxalone in marmosets. The aim was to find a safe, reliable, short-term sedation protocol, with at least 10 min of immobilisation. In this study, alphaxalone was shown to be superior for sedation in marmosets. Due to the relatively long recovery period, ketamine-medetomidine is not the first choice in the used dose and dosing regime for sedation in marmosets.

All induction times were less than 5 min. Induction time, defined as the time between injection and loss of postural tonicity, was a very accurate measure since the transition from “awake” to “immobilised” was very distinct.

The duration of immobilisation indicated that the alphaxalone dosage could be used safely for procedures lasting approximately 40 min. Additionally, all marmosets displayed muscle twitches near the end of immobilisation. Alphaxalone is described in cats and dogs to cause myoclonic twitches [14]. In marmosets we observed these muscle twitches only just before awakening, which could be interpreted as 'warning of awakening’ signs towards the end of the immobilisation period. The shorter duration of immobilisation of the other protocols demonstrate that those protocols can only be used safely for procedures lasting less than 15 min. Notably, in protocol 3A and 3B, atipamezole was administered after 10 minutes of immobilisation, thus in this study we can not report on the duration of immobilisation in these 2 protocols when atipamezole was administered later or not at all.

In our initial set-up, we found an undesirable long recovery time in protocol 3A, despite the administration of atipamezole. This was surprising, as it was expected to be shorter than for all other sedatives due to the administration of a specific antagonist of medetomidine: atipamezole. Short total procedure times are preferred as it minimises the time between the animal’s removal from and return to its social group, and gives the advantage of the animal being able to consume food rapidly post sedation. In cats and dogs, reversal with atipamezole results in a quick and full recovery (3–5 min) [23,24]. This combination is also described as a common induction regime for non-human primates [3]. Our observations show the opposite in marmosets, and are in line with a study by Young [35], who found no difference in recovery times between macaques that received ketamine-medetomidine reversed with atipamezole compared to ketamine only. Although ketamine supplemented with a lower dose of medetomidine and an additional atipamezole injection tended to result in a shorter recovery time compared to the high medetomidine dose group, the recovery duration remained protracted and unacceptable. At the moment, we have no explanation for this finding. Particularly in protocol 3A and 3B, after an initial arousal involving sitting upright and an attempt to climb, the animals spent several hours in complete apathy. During this period of apathy it was not safe to reunite them with their companion, as marmosets engaged in a conflict of dominance when one was temporarily not fully awake. This demonstrates that recovery should be well defined and is not merely waking-up after sedation.

The drug dosages were chosen according to institute-wide practices and published references [37,38]. Our experience with the used protocols show that they were sufficient for minor invasive procedures, such as blood collection from the femoral vein, tuberculin testing, wound care, and radiography (data not shown). The observed duration times showed that the doses and/or use of antagonists were well chosen, as induction time for all protocols was very short, and immobilisation times, without taking protocol 2 into account, were not long enough to allow a dose reduction without increasing the risk of creating a immobilisation period shorter than 10 min; our dosage can only be used for minimal procedures requiring less than 15 min, and maybe even then a loss of reliability can occur.

Considering the overall quality of the different protocols tested, sedation using alphaxalone shows to be the most optimal as we observed no (excessive) salivation, apnoea, involuntary muscle movement, or vomiting for this protocol. The side effects we have observed with ketamine in protocol 1 are consistent with literature [4-6]. Retching and vomiting during recovery, as seen in protocol 3A and 3B, is known to be a common side effect of α2-adrenergic agonists [39]. However, retching and vomiting were not seen in macaques given ketamine-medetomidine [35]. The observed retching and vomiting during recovery, could also be due atipamezole, however, no information is available about the side effects of atipamezole in primates [28-30,32-35].

The most marked effect on physiological parameters was hypothermia, which probably delayed recovery from sedation in all animals in all sedation protocols, but all recoveries were uneventful and no long-term side effects were observed. Nevertheless, the use of a heating pad or a lamp would be beneficial and should always be used during sedation and recovery, as described for macaques [40].

The limited changes in PR, MAP, RR and %SpO_2_ in all protocols remained within a clinically acceptable range in most animals, with the exception of two animals in which apnoea was scored during protocol 1 and one also during protocol 3B between the atipamezole injections, which suggests that the apnoeae were a ketamine side-effect. Nevertheless, the recovery times of both animals were not prolonged compared to the other animals in the same protocol and the animals recovered without intervention. In conscious unrestrained marmosets an PR of 230 ± 26 bpm is described [41]. The small drop in PR and MAP observed at the start of the sedation procedure (Table 6) was possibly due to a deepening of the plane of sedation, as the drugs were absorbed from the injection site - and rose in the end. In dogs and cats, bradycardia is consistently seen with the use of medetomidine due to a combination of central reduction in the sympathetic drive to the heart and reflex bradycardia following peripheral vasoconstriction [23,24,42], and can also cause respiratory depression [34]. In marmosets there was no significant difference observed in blood pressure drop between the ketamine and both ketamine-medetomidine groups. The decrease in PR is likely to be central in origin, although an initial transient hypertension would probably not have been detected. In the current study, the marmosets sedated with alphaxalone had a lower, although not significantly, RR when compared to the other sedation protocols, however not significant. This lower RR for alphaxalone is also described in dogs and cats [12,14]. However, the %SpO_2_ values did not differ significantly between the protocols. The recorded %SpO_2_ levels were lower than the generally accepted minimum of 95%, which indicates a certain level of hypoxia. However, the recorded %SpO_2_ may have been not reliable due to a bias caused by the peripheral vasoconstriction effect of medetomidine or due pigment interference with the sensor’s capacity to read accurately [Feiner et al. 2007 [43]].

The withdrawal reaction and palpebral reflex, together with muscle tension, were used to determine the levels of sedation and analgesia in the present study [29,36]. However, some anaesthetics not only sedate animals and produce analgesia but they also interfere with the responses used to measure these conditions. Ketamine is known to induce deep sedation without reducing the palpebral reflex [36,44]. In contrast, in animals sedated with alphaxalone, there is a reduced palpebral reflex suggesting that this anaesthetic does not interfere with this response. In the present study, alphaxalone induced the deepest sedation and analgesia as measured by these responses. No further literature is available regarding the analgesic effects of alphaxalone in marmosets.

In addition, as described in other studies [32,35], a combination of medetomide and ketamine provides more muscle relaxation than ketamine alone.

Increased AST, LDH, and CK levels in protocol 1 were indicative for local myotoxicity of the injected formulation. These results are in accordance to published data on local myotoxicity of the injected formulations in marmosets and other primates [7-9]. Protocol 2 is preferred in marmosets, as it did not cause muscle damage as indicated by the lack of increase in AST, LDH and CK values, despite the relatively large injection volume. This is in according to literature about the use of Saffan in primates [5].

Bodyweight loss was highest in protocol 3A compared to the other protocols, explainable by the fact the animals had a much longer recovery time in which they were not able or willing to eat. The difference between protocol 3A and 3B shows that sedative dosages need to be chosen well as small dose changes indirectly influence important parameters as bodyweight.

## Conclusion

The aim of this study was to find a good, short-term sedation protocol, with at least 10 min of immobilisation. Our finding that alphaxolone showed the shortest total procedure time combined with the longest immobilisation time demonstrate that alphaxalone has major practical advantages over the other sedative protocols tested.

## Competing interests

The authors confirm that they have no competing interests in the conduct of this research or preparation of this paper.

## Authors’ contributions

JB, JJU, HPMB, and JAML conceived the study and participated in its design and coordination and wrote the final version of the manuscript. ERJP collected data and EJR performed statistical analysis and interpreted the data obtained. All authors have read and approved the final manuscript.
